# Peri-implantitis and heart rate variability: a missing modifiable risk determinant

**DOI:** 10.3389/fdmed.2026.1964007

**Published:** 2026-10-20

**Authors:** David Cheng

**Affiliations:** Westcoast Smile Dental Studio and Whitening Spa, Vancouver, BC, Canada

**Keywords:** autonomic nervous system, cholinergic anti-inflammatory pathway, heart rate variability, host susceptibility, implant failure, peri-implant bone loss, peri-implantitis, vagal tone

## Abstract

This review proposes heart rate variability, a non-invasive index of autonomic regulatory capacity, as a candidate modifiable systemic determinant of peri-implantitis. Peri-implantitis, a bone-destructive complication affecting roughly one in five implant patients, has no validated prevention model. Despite four decades of implant design and surface-engineering research, the most recent consensus synthesis identified risk indicators but no confirmed risk factors. This review argues that the difficulty is structural: much of the design literature has been evaluated against marginal bone loss, an unvalidated radiographic surrogate for the disease, while no modifiable systemic determinant of host capacity has yet been established. Three observations are developed from the published literature. First, early marginal bone loss is substantially physiological, reflecting biologic-width formation; design factors that reduce it, including platform switching and connection geometry, have not been shown to reduce the disease itself. Second, well-measured risk indicators carry effect sizes consistent with modifiers rather than causes, and the literature repeatedly reports synergy rather than addition, the behavior of a gated rather than an additive system. Third, the proposed mechanisms converge on a shared effector, macrophage activation with NF-*κ*B signaling and a RANKL-dominant RANKL/OPG ratio. The most extensively studied inherited host-susceptibility candidate, the interleukin-1 genotype, is fixed and unmodifiable. While the sympathetic and hypothalamic-pituitary-adrenal stress axis has been documented in peri-implant and periodontal disease, the counter-regulatory parasympathetic arm, indexed by heart rate variability, has not yet been examined as a modifiable determinant. Its anti-inflammatory arm is proposed to act through the cholinergic anti-inflammatory pathway; the review presents a two-level model in which systemic capacity sets the disease threshold while local load determines which sites reach it. Peri-implantitis may be usefully reframed as a disorder of host regulatory capacity acting on a shared inflammatory effector, rather than as a disease driven by any single local load. This hypothesis generates specific, falsifiable predictions, identifies heart rate variability as a testable biomarker, and would relocate prevention from the implant surface to the regulatory state of the host. The immediate priority is a prospective study relating baseline heart rate variability to peri-implantitis incidence.

## Introduction

### The clinician's problem

Dental implants succeed reliably, yet a substantial minority of patients develop peri-implantitis, a progressive inflammatory destruction of the bone that supports an osseointegrated implant. Recent pooled data from Galarraga-Vinueza et al. place its prevalence near 21 percent at the patient level, with an incidence of roughly 22 percent over twenty years (1). The most recent and largest synthesis of the evidence, a 2025 consensus spanning 102 studies and more than 13,000 patients, identified risk indicators but no confirmed risk factors for peri-implantitis (1). The difficulty is therefore structural, not merely incomplete. For the clinician it is not that the disease occurs, but that it cannot be predicted, prevented, or reliably arrested. The available guidance to patients is largely non-specific or, on the evidence, inconclusive: stop smoking, control plaque, attend maintenance. When a patient develops peri-implantitis despite following that guidance, or avoids it despite ignoring it, the clinician has no mechanistic account to offer.

An implant may fail mechanically, through screw loosening, component fracture, or prosthetic complication. It may fail to osseointegrate, an early event reflecting the biology of initial healing. Or it may lose supporting bone after successful integration, through the inflammatory disease that is peri-implantitis. These are different phenomena with different mechanisms and different timelines. This review concerns only the last. Marginal bone loss, implant failure, and peri-implantitis are not interchangeable endpoints, and much of the confusion in the risk-factor literature originates in treating them as if they were.

### Four scenarios that resist a single-cause explanation

Certain clinical patterns occur. No single one refutes a local-factor model, but each constrains what an adequate model must explain, and together they are difficult to account for within a purely local one.

The first is symmetry. Peri-implantitis is sometimes encountered in a symmetric distribution, affecting contralateral implants, for example at the mandibular left and right first molar sites, with near-identical patterns and severity of bone loss. On its own, symmetry does not require a systemic explanation, since contralateral sites often carry mirror-image local loads: comparable occlusal scheme, crown contour, and access for hygiene. What remains unexplained is the near-identical severity of the two lesions, a concordance between contralateral sites that has not been examined.

The second is selectivity. A patient may carry several implants placed at the same visit by the same operator, of which only one or two develop disease while the others remain healthy for years, under the same systemic conditions and the same oral environment.

The third is the probabilistic behavior of the strongest behavioral risk factor. Smoking is consistently associated with implant complications, yet meta-analysis places the effect on implant failure at an odds ratio near two (2), and the most recent consensus rates smoking as only a modest risk indicator for peri-implantitis specifically (1). An effect of that magnitude means most implants in smokers do not develop the disease. Smoking raises probability; it does not determine outcome.

The fourth is the occurrence of peri-implantitis in patients with none of the recognized risk indicators. In a referral population studied by Serino and Strom, many patients presenting with peri-implantitis had healthy periodontium and did not smoke (3).

Taken together, symmetry, selectivity, probabilistic risk, and disease in the absence of risk indicators point to a variable that is neither purely local nor captured by the current model. This review develops an account of what that variable may be. It proceeds deliberately: first by showing that much of what the field measures is not the disease, then by showing that what does predict the disease behaves like a modifier rather than a cause, then by identifying the class of variable the evidence points toward, and only then by proposing a specific candidate within that class.

## Search strategy

This is a narrative review with an explicit hypothesis. The literature was searched in PubMed, Scopus, and Web of Science from inception through 2026, in English, across four domains: peri-implantitis etiology, risk indicators, and epidemiology; the physiology of peri-implant and biologic-width bone remodeling; periprosthetic osteolysis and the macrophage-NF-*κ*B-RANKL effector; and autonomic regulation of inflammation and bone, including the cholinergic anti-inflammatory pathway and heart rate variability. A bridging search combining peri-implantitis with autonomic, vagal, cholinergic, and heart-rate-variability terms was performed to establish whether the central hypothesis had been examined previously. Studies were selected for relevance to mechanism, endpoint validity, or effect size. Reference lists of key reviews were hand-searched. Evidence was synthesized narratively; no quantitative pooling was performed. Because this is a hypothesis-driven narrative review rather than a systematic review, no formal PRISMA-style inclusion and exclusion framework was applied; studies that did not materially inform the proposed mechanism, endpoint validity, effect size, or testable predictions were not included. Importantly, the bridging search identified no direct prospective studies evaluating heart rate variability or parasympathetic regulatory capacity as determinants of peri-implantitis; the hypothesis therefore necessarily draws on convergent evidence from adjacent fields.

## Part 1. The measurement problem: physiological versus pathological bone loss

The endpoint used to evaluate almost every implant design decision is marginal bone loss, a continuous radiographic measurement. The endpoint that matters clinically is peri-implantitis, a diagnosed inflammatory disease. These are not the same, and the gap between them is the foundation of this review.

Early crestal bone remodeling following implant exposure is primarily a physiological response to supracrestal tissue attachment formation rather than a pathological process. When an implant is exposed to the oral environment, the peri-implant tissues establish a biologic width, now termed supracrestal tissue attachment, an epithelial and connective-tissue attachment of roughly two to three millimeters, and where the crestal bone lies coronal to the level this dimension requires, it remodels away to accommodate it, as described by Farronato et al. (4). The magnitude of this remodeling is determined substantially by mucosal thickness. In a within-patient comparison, implants placed in thin tissue lost markedly more crestal bone than those placed with adequate vertical soft tissue or positioned subcrestally, the difference approaching 0.7 millimeters and established by the time the restoration was delivered (5). Linkevicius et al. showed that managing the biologic-width requirement, by increasing tissue thickness or by subcrestal placement, largely reduces this early loss (6). This is not the prevention of a disease. It is the accommodation of anatomical dimension.

The consequence is that a given measurement of early crestal bone loss carries two different meanings that a radiograph cannot distinguish. It may represent benign, self-limiting biologic-width formation, or it may represent the opening phase of a pathological process. Whether early bone loss predicts later disease has been examined directly only in prospective cohorts. In a ten-year prospective cohort study by Windael et al., implant survival was 94.74 percent, and mean crestal bone loss at ten years was 0.81 millimeters (7). Early loss did flag higher risk, but the signal it gives is a quantity of bone already lost, the surrogate, not the onset of disease.

The surrogate's usefulness is further limited by a detection threshold. Conventional radiographs cannot detect early bone loss until sufficient mineral loss has occurred to become radiographically visible; a peri-implant radiolucency reflects demineralization that has crossed a threshold of visibility, not the onset of loss itself (8). Below that threshold, bone resorption may already be underway while the radiograph reads as normal. One early-warning signal has been demonstrated: bone loss exceeding 0.5 millimeters within the first year of function raises the odds of later peri-implantitis more than fivefold in a ten-year prospective cohort (7). But this finding still depends on bone loss having become radiographically measurable; it narrows the window; it does not close it. At present, no validated method exists to identify peri-implantitis before it is radiographically and clinically apparent. This is not a gap adjacent to the measurement problem described above. It is the same gap, restated from the standpoint of detection rather than interpretation.

This distinction reframes a large body of design research, which is examined next.

## Part 2. The sieve: design factors predict the surrogate, not the disease

If marginal bone loss is substantially physiological, then interventions that reduce it may be managing biologic-width remodeling rather than preventing peri-implantitis. The evidence indicates that this is largely the case.

Platform switching, the horizontal relocation of the implant-abutment junction inward from the implant periphery, is one of the most extensively studied implant-abutment design modifications. Meta-analyses of randomized trials, including work by Mishra et al., show a significant pooled reduction in marginal bone loss of roughly 0.7 millimeters with platform switching, with greater mismatch generally associated with greater preservation of marginal bone (9), and in a systematic review, Tajti et al. found that bone-level implants restored with long abutments showed less marginal bone loss (10). Yet when peri-implantitis itself is the endpoint, the effect is not demonstrable. In a cross-sectional pilot comparison by Duque et al. that directly assessed peri-implant disease, platform switching produced only a non-significant trend toward lower peri-implantitis prevalence (6.6% versus 15.6%), while peri-implant mucositis was numerically higher in the platform-switching group (11).

The same pattern holds across the design variables. Connection type influences crestal bone loss, with conical Morse-taper connections outperforming external-hex designs and leaking less at the interface, but the network meta-analysis by Caricasulo et al. and the microleakage systematic review by Mishra et al. show that these advantages are confounded with platform-switching geometry and have not been demonstrated to translate into reduced peri-implantitis (12, 13). The microgap, the source of fretting and microleakage at the junction, is a plausible mechanistic contributor, yet eliminating it entirely by using one-piece implants has not been shown to reduce peri-implantitis relative to two-piece designs; the available meta-analysis by Liu et al. found no significant difference in biological complications, and two-piece implants with platform switching actually showed marginally less bone loss than one-piece implants (14). Placement depth and mucosal thickness influence early bone loss through the biologic-width mechanism described above, not through disease prevention (5, 6). Implant surface has been examined extensively, and the Monje et al. 2025 AO/AAP consensus synthesis of implant-related factors concluded that comparative clinical studies have not demonstrated consistent superiority for any implant macro- or micro-design characteristic, including surface characteristics (15). Where a surface signal does appear, it concerns the progression of already-initiated disease, with rougher surfaces tending to accelerate progression in preclinical models, rather than evidence that surface selection prevents peri-implantitis (15). Implant material and geometry alter the quantity and composition of titanium particle release, with alloys shedding more than commercially pure titanium and wider or cylindrical designs shedding more than narrow or tapered ones, but as shown by the *ex vivo* work of Barrak et al. and the review by Chen et al., current evidence has not established that these differences in particle burden translate into differences in peri-implantitis risk (16, 17). One caution applies across these comparisons. In most studies, peri-implantitis is not the primary endpoint; marginal bone loss is, and studies adequately powered to detect differences in peri-implantitis are relatively few. The repeated absence of a disease-level effect therefore reflects, in part, how infrequently peri-implantitis has been directly evaluated, not simply that these factors have no effect on the disease.

The consistent finding is that the variables clinicians’ control at implant placement—including connection type, platform design, placement depth, surface characteristics, material, and geometry—influence the radiographic surrogate but have not been shown to consistently influence peri-implantitis. This is not evidence that these variables are irrelevant to marginal bone levels. Rather, it indicates that reducing marginal bone loss cannot be assumed to reduce peri-implantitis, consistent with the conclusion of Part 1 that the surrogate and the disease are not the same.

Emerging implant technologies further illustrate that implant design may actively modify the local biological environment. Chen et al. recently developed an occlusion-activated piezoelectric implant that converted physiological loading into electrical signals and demonstrated antibacterial activity through controlled reactive oxygen species generation, modulation of macrophages toward an anti-inflammatory phenotype, and enhanced osteogenesis in preclinical models (18). These findings do not yet establish clinical prevention of peri-implantitis but demonstrate that advanced implant engineering can influence local inflammatory and bone-remodeling pathways. Such approaches are complementary to the present two-level model, in which implant-related factors modify local load and tissue response while systemic autonomic capacity may influence the host threshold for containing that challenge.

## Part 3. What does predict the disease behave like a modifier, not a cause

The factors that do show association with peri-implantitis as a diagnosed entity are, with few exceptions, host-level and site-level rather than design-level, and their effect sizes are informative.

History of periodontitis is the most consistent predictor, with a pooled odds ratio near 2.9, rising to roughly 4.0 for active periodontitis, and reflects a host characteristic: individuals susceptible to periodontal breakdown remain susceptible to peri-implantitis after rehabilitation with implants (1, 19).

Absence of an adequate band of keratinized mucosa is associated with peri-implantitis (pooled OR approximately 2.8), with studies adjusting for confounding variables reporting a pooled OR of 3.68 (20). The interleukin-1 composite genotype, the field's most extensively studied inherited host-susceptibility candidate, carries an odds ratio of approximately 2.0, although the estimate is based on a limited evidence base. The meta-analysis by Jin et al. reported this association but emphasized the need for larger, higher-quality studies to confirm it (21), and the meta-analysis by Mohammadi et al., applying Trial Sequential Analysis, found that the available evidence had not reached the required information size to confirm the association (22). The five-year cohort study by Costa et al. found that absence of supportive maintenance was associated with an approximately 2.4-fold higher incidence of peri-implantitis (23). Diabetes carries a significant but non-causal association with peri-implantitis (OR approximately 2.3), and smoking a similarly modest one, consistent with risk indicators rather than confirmed risk factors (1). Occlusal overload, long of uncertain significance, was recently associated with peri-implantitis in a systematic review by Mojaver et al., but explicitly in synergy with biofilm rather than as an independent cause (24). Corbella et al. identified a prosthetic emergence-profile angle of 45 degrees or more as a significant contributor to peri-implantitis risk (OR 9.1) (25), although Romandini et al., testing the same factor in an independent cohort, did not find a significant association (19).

Two features of this list matter more than any individual entry. First, the replicated host- and systemic-level predictors cluster in a relatively narrow range. The most consistently reported and well-characterized associations generally fall between odds ratios of approximately 2 and 4, including history of periodontitis, absence of keratinized mucosa, and lack of supportive maintenance. These effect sizes are more consistent with modifiers of host susceptibility than with sufficient causes of disease. Larger odds ratios have been reported for some local factors. Close inter-implant distance, for example, was associated with a substantially higher odds ratio in the same systematic review (15), although this estimate was derived from limited single-study evidence with wide confidence intervals and has not been consistently replicated. Its proposed mechanism is site-specific—restricted interproximal hygiene access and altered local tissue conditions—rather than a systemic determinant. Similarly, prosthetic emergence profile has been associated with peri-implantitis at the implant level (25). Collectively, these local factors describe where biomechanical and inflammatory burden may concentrate, rather than what determines the host response to it. Accordingly, the two-level model developed below distinguishes between local load, which may vary substantially between implant sites, and systemic modifiers, which act across all sites within the same individual.

Second, the literature repeatedly describes interactions rather than simple independent effects. Examples include occlusal overload acting in conjunction with biofilm (24) and titanium ions interacting with lipopolysaccharide to increase the RANKL/OPG ratio (17). These observations are not fully captured by models in which risk indicators act independently (Figure 1). The threshold model proposed here provides one possible framework for explaining such interactions.

**Figure 1:**
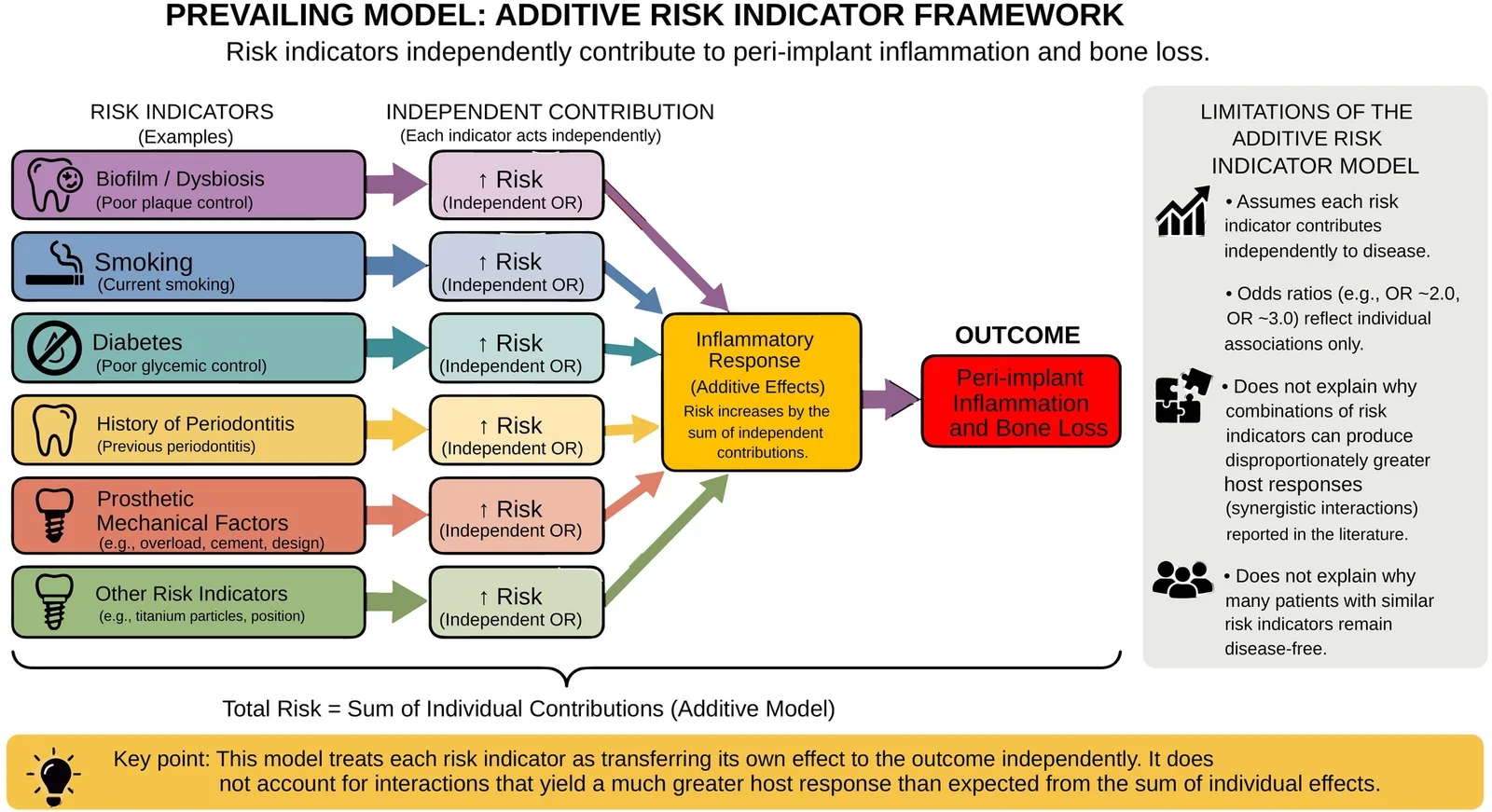
The prevailing model, in which risk indicators feed additively into a single linear chain. Six local and systemic risk indicators, biofilm, smoking, diabetes, history of periodontitis, prosthetic and mechanical factors, and other local factors, are each treated as independent contributors that add to a common inflammatory response and produce peri-implant bone loss. The model contains no term for the host's capacity to restrain the response, which has three consequences developed in this review: risk indicators are undifferentiated by whether they act locally at a site or systemically across the host; each indicator is expected to resolve into an independent effect, which is difficult to reconcile with the probabilistic behavior of the evidence and the synergy repeatedly reported in the literature; and the model offers no mechanistic route to prevention for the patient without identifiable risk indicators.

This pattern is reinforced at the highest level of synthesis. The consensus finding introduced above—that only risk indicators could be identified across 102 studies and more than 13,000 patients (1)—raises the possibility that an important class of host-level regulatory variables has not yet been routinely measured. This interpretation does not diminish the importance of established local and systemic risk indicators; rather, it suggests that they may operate within a broader regulatory framework that has yet to be fully characterized.

These modifiers do not act on an inert scaffold. Peri-implant bone is an actively regulated, mechanosensitive tissue, and, in the absence of the periodontal ligament, its remodeling is mediated through the RANKL/OPG signaling pathway, onto which local mechanical loading and, as proposed below, systemic neural regulation may converge. Peri-implantitis therefore develops within a dynamically regulated biological tissue rather than at a passive site of injury. This provides the rationale for examining whether systemic physiological regulation contributes to an individual's susceptibility to peri-implant disease.

## Part 4. Convergence on a shared effector

Beneath the heterogeneity of proposed factors lies a striking uniformity of mechanism. Despite diverse initiating stimuli, peri-implant bone destruction converges on a common downstream effector pathway: activation of macrophages, NF-*κ*B signaling, production of the pro-inflammatory cytokines IL-1β, TNF-α and IL-6, and a shift in the RANKL/RANK/OPG axis toward osteoclastogenesis and bone resorption (17, 26).

This same downstream inflammatory pathway has been established in orthopedic periprosthetic osteolysis, where wear particles activate macrophages through innate immune signaling, leading to NF-*κ*B activation, a shift in the RANKL/OPG axis, and osteoclast-mediated bone resorption (26, 27). It is also the pathway engaged by titanium particles in peri-implant tissues (17). The same host-response logic is now central to understanding periodontitis itself, in which disease emerges from polymicrobial dysbiosis interacting with a dysregulated inflammatory response in a susceptible individual, rather than from a single pathogen acting alone (28). Greater vulnerability of peri-implant than periodontal tissues to a comparable microbial challenge was demonstrated experimentally by Heyman et al.: in the same animals, infection produced a markedly greater influx of leukocytes in the peri-implant mucosa than in gingiva around teeth, while increasing the local RANKL/OPG ratio; experimental peri-implant bone loss was prevented by resolving the host inflammatory response with Resolvin D2 (29).

Microbial challenge is not the only trigger capable of engaging this pathway. Titanium particles and dissolved titanium are commonly detected around dental implants. They may be released during implant placement and function, with their quantity and composition influenced by implant material, geometry, surface characteristics, corrosion, and mechanical manipulation (16). Titanium has been detected around both clinically healthy and diseased implants, although higher levels have been reported at peri-implantitis sites (17, 30). The review by Chen et al. concluded that, although titanium particles are biologically active and can amplify inflammatory responses, their presence alone does not explain why some sites progress to peri-implantitis (17). The same AO/AAP consensus panel did not reach consensus on whether titanium biomaterial degradation contributes to peri-implant disease, with only a simple majority supporting the proposition (31). This is consistent with titanium particle burden being biologically relevant but insufficient, by itself, to distinguish health from disease. The discriminating factor may therefore be not whether the inflammatory effector pathway is engaged, but whether the host contains and resolves the response.

## Part 5. A missing class of variable

Assemble the evidence and a specific absence becomes visible.

The disease is host-governed, converging on a regulable inflammatory effector pathway. Susceptibility varies between individuals. Yet the field's most studied inherited host-susceptibility candidate, the interleukin-1 composite genotype, is fixed at birth, unmodifiable, and confers only a modest increase in risk, while the available evidence remains insufficient for firm confirmation (21, 22). At present, no validated, modifiable, measurable systemic marker of host capacity has been identified in the peri-implantitis literature.

There is, moreover, a striking asymmetry in what has been examined. The autonomic nervous system comprises two functional arms. The sympathetic arm, together with the hypothalamic-pituitary-adrenal axis, mediates the physiological stress response by increasing catecholamines and cortisol and has therefore been the principal focus of research in periodontal and peri-implant disease. Depression, the chronic psychological stress subtype most consistently associated with peri-implantitis, has been proposed as the strongest psychological correlate of disease, with peri-implant sulcus cortisol levels correlating with probing depth (32). A recent meta-analysis similarly found elevated salivary cortisol levels in patients with periodontitis (33), while Decker et al. reviewed the psychobiological pathways through which chronic stress may contribute to periodontal and peri-implant disease progression (34). Salivary *α*-amylase, a surrogate marker of sympathetic activity, has also been investigated together with inflammatory cytokines in peri-implantitis patients (35). By contrast, the parasympathetic arm has received remarkably little attention. Where heart rate variability has entered the periodontal literature, it has generally been used to examine autonomic changes accompanying established periodontal disease rather than as a prospective measure of host susceptibility. For example, Ribeiro et al. investigated cardiovascular autonomic regulation after induction of experimental periodontitis in hypertensive and normotensive rats, but did not evaluate baseline heart rate variability as a predictor of periodontal destruction (36).

The parasympathetic arm has not received comparable attention. Vagal cholinergic signaling can restrain macrophage cytokine production through the cholinergic anti-inflammatory pathway, yet this counter-regulatory mechanism has not been directly measured in peri-implantitis, in human periodontitis as a determinant of bone loss, or in implant cohorts. The accelerator has been documented; the brake has not been examined in the same way. The same consensus panel classified depression and antidepressant use as a risk indicator for peri-implantitis with 93% agreement (31). Major depression is itself associated with reduced heart rate variability, including reductions in vagally mediated indices (37). A correlate of diminished parasympathetic regulation may therefore already have appeared among the field's risk indicators, although this connection remains inferential and is offered as consistency rather than evidence.

This absence is not unique to autonomic literature. The peri-implantitis biomarker literature more broadly, including matrix metalloproteinase-8 (MMP-8), its activated form (aMMP-8), interleukin-1β, and the soluble RANKL/OPG ratio, has established diagnostic and treatment-monitoring value (38–40). These biomarkers reliably distinguish diseased from healthy implants at a single point in time, and some have been shown to track resolution following treatment. However, none has been prospectively validated as a modifiable measure of host susceptibility or host regulatory capacity. Likewise, no study has measured these biomarkers, or systemic stress markers such as salivary cortisol or salivary *α*-amylase, in disease-free implant patients and followed them longitudinally to determine who subsequently develops peri-implantitis. The literature establishes what changes once disease is present; it does not yet establish what precedes disease onset. Heart rate variability shares this same prospective evidence gap rather than being uniquely burdened by it. The proposed study in Part 8 is designed to address that gap directly.

What remains missing is a measurable, modifiable systemic variable that reflects host regulatory capacity before clinical disease develops. The available evidence suggests that this gap is particularly evident for the parasympathetic counter-regulatory system, which has received comparatively little attention in peri-implant research. The remainder of this review proposes heart rate variability as a candidate measure of that host capacity.

## Part 6. The candidate: autonomic regulatory capacity and the cholinergic anti-inflammatory pathway

The vagus nerve balances innate immune activation through the cholinergic anti-inflammatory pathway, in which acetylcholine acting on nicotinic receptors suppresses macrophage production of pro-inflammatory cytokines including TNF-α, IL-1β, and IL-6. This neural regulation of inflammation forms the basis of the inflammatory reflex first described by Tracey (41). Because these cytokines converge on the inflammatory pathways responsible for peri-implant bone resorption, this mechanism provides a biologically plausible link between systemic neural regulation and local peri-implant tissue destruction. The activity of this pathway is thought to reflect vagal function. Cardiac parasympathetic modulation can be estimated non-invasively using vagally mediated heart rate variability indices, although heart rate variability reflects cardiac autonomic regulation rather than directly measuring vagal signaling to macrophages or other immune tissues (42).

The absence of the periodontal ligament is an important anatomical distinction between natural teeth and osseointegrated implants. Tooth extraction removes the periodontal mechanoreceptor apparatus and alters the local neural environment; however, peri-implant tissues undergo reinnervation, with nerve fibers demonstrated experimentally and histologically within peri-implant tissues and bone (43, 44). Sympathetic regulation also remains functionally relevant in the absence of a periodontal ligament, as experimental sympathectomy alters peri-implant bone formation and osseointegration (45). More broadly, skeletal neurobiology supports both local and systemic neural regulation of bone, whereas direct parasympathetic innervation of mature peri-implant bone remains less clearly established (46).

This distinction is particularly relevant to the cholinergic anti-inflammatory pathway. Direct parasympathetic axonal contact with the implant-bone interface is not necessarily required for cholinergic immunoregulation. Autonomic signals can be relayed through neuroimmune pathways in which acetylcholine-producing immune cells provide local cholinergic signaling; ChAT-positive T cells, for example, can relay vagal signals through acetylcholine release (47). Acetylcholine can then act through *α*7 nicotinic acetylcholine receptors on macrophages to regulate inflammatory cytokine production (48), while non-neuronal cholinergic systems allow immune and other peripheral cells to synthesize and respond to acetylcholine through local autocrine and paracrine signaling (49). A functional non-neuronal cholinergic system has also been proposed within periodontal tissues (50). Thus, loss of the periodontal ligament may alter local neural regulation without necessarily abolishing cholinergic immunomodulation or systemic autonomic influence. Whether these mechanisms operate specifically at the human peri-implant bone interface remains to be established.

The relevance of this pathway to bone is not merely theoretical, although it remains untested in peri-implantitis. In experimental periodontitis, Xie et al. demonstrated that vagus nerve stimulation reduced alveolar bone loss, suppressed local inflammatory cytokine expression, and activated the *α*7 nicotinic acetylcholine receptor pathway (51). Similarly, Ribeiro et al. showed that carotid sinus nerve stimulation attenuated alveolar bone loss and periodontal inflammation through autonomic reflex activation (52). Beyond periodontal disease, the parasympathetic nervous system has also been shown to regulate skeletal remodeling directly. Bajayo et al. demonstrated that parasympathetic signaling regulates bone mass by modulating osteoclast activity through nicotinic receptor signaling (53). This broader concept of neural regulation of the jawbone is reflected in a 2023 review by Wu et al. which summarizes evidence that sympathetic signaling promotes RANKL-mediated bone resorption, whereas parasympathetic signaling contributes to anti-inflammatory regulation and osteoclast apoptosis. Importantly, the authors conclude that the effects of acetylcholine on jawbone remodeling remain insufficiently understood (54). Thus, the gap proposed here has already been identified within the oral biology literature itself.

The most complete demonstration that this axis operates in a chronic, bone-destructive, RANKL-mediated inflammatory disease comes from rheumatoid arthritis, which differs from peri-implantitis in etiology but shares key downstream inflammatory and osteoclastogenic pathways. In individuals at risk for rheumatoid arthritis, autonomic dysfunction has been shown to precede and predict disease onset; in established disease, higher vagal tone measured by heart rate variability is associated with better treatment response, and vagus nerve stimulation lowers TNF production and improves clinical disease activity in humans (55, 56). Preclinical arthritis studies summarized by Koopman et al. further show that deletion of the *α*7 nicotinic acetylcholine receptor worsens inflammatory arthritis (56). By contrast, deletion of the same receptor increases bone mass in otherwise unchallenged animals by raising the OPG/RANKL ratio and reducing osteoclast formation (57). This context dependence must be stated plainly: the effects of cholinergic signaling on bone differ between the quiescent and inflamed states. Peri-implantitis is an inflammatory, bone-destructive state, for which the periodontal and arthritis models above are the more appropriate analogues.

The hypothesis of this review follows directly from the preceding evidence. Autonomic regulatory capacity, specifically the parasympathetic counter-regulatory reserve indexed by heart rate variability, is proposed as a modifiable systemic determinant of whether the peri-implant foreign-body challenge is contained or progresses to disease. A bridging literature search identified no prior study investigating parasympathetic regulatory capacity, vagal tone, or heart rate variability as a modifiable determinant of peri-implantitis. The autonomic nervous system has been discussed only in broad terms within the peri-implant literature. For example, Ball and Darby identified autonomic regulation as one of several biological pathways linking mental health and peri-implant disease but did not develop the cholinergic anti-inflammatory pathway, vagal regulatory capacity, or heart rate variability as a measurable host-susceptibility variable (58). To the best of the author's knowledge, the specific hypothesis proposed here has not previously been advanced.

## Part 7. A two-level model

The proposition resolves into a two-level model (Figure 2), which provides a unified explanation for the clinical scenarios that opened this review.

**Figure 2:**
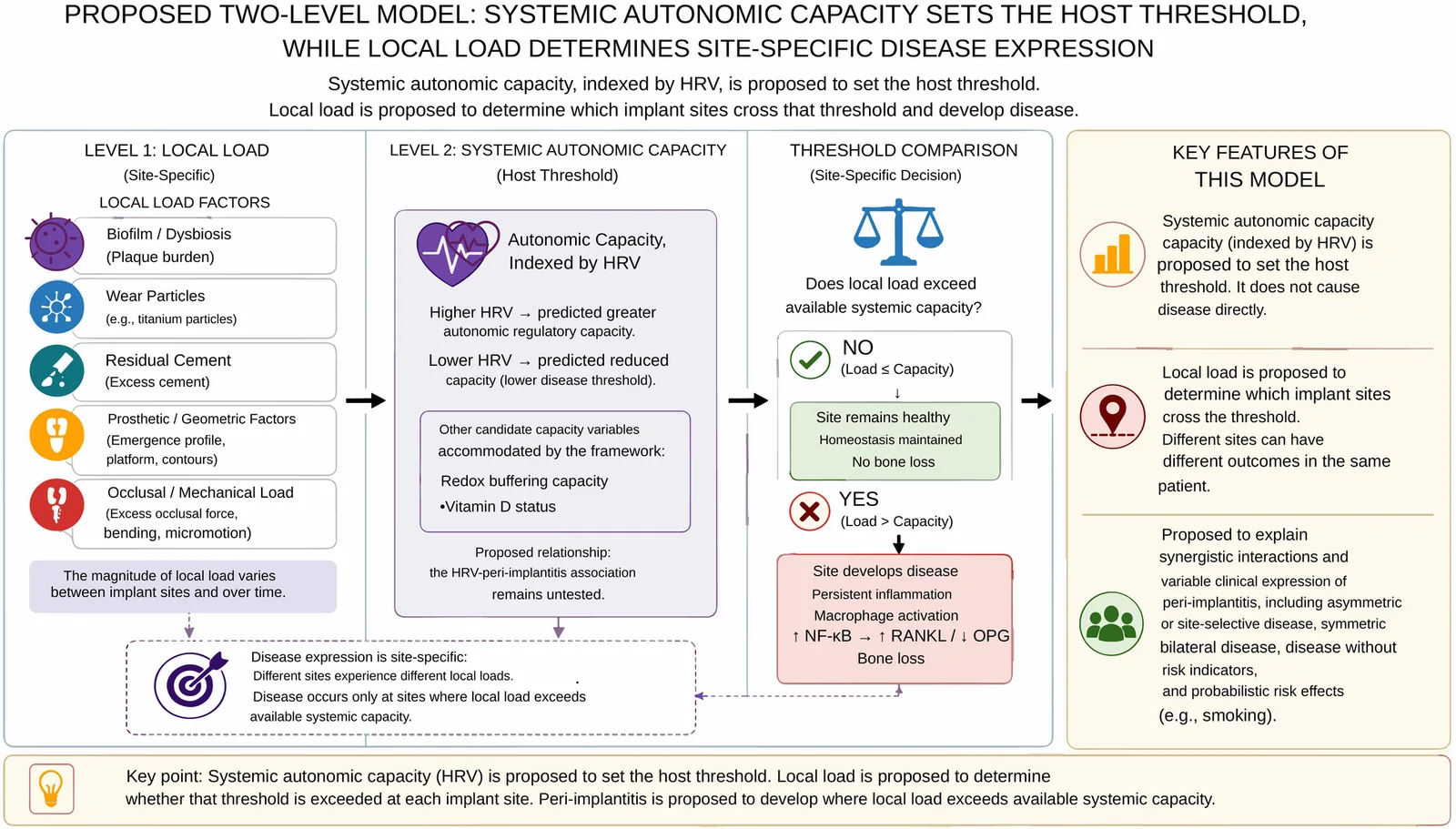
Proposed two-level model: local load drives, and systemic autonomic capacity restrains, a shared effector. The effector is unchanged from Figure 1. Local load is site-specific, varies between implant sites within a patient, and comprises biofilm, wear particles, residual cement, prosthetic and geometric factors, advanced design/manufacturing characteristics, and occlusal load. Advanced design/manufacturing characteristics may modify local mechanical or biological burden rather than necessarily increase it. Systemic autonomic capacity is patient-specific, reflects parasympathetic reserve whose anti-inflammatory arm is proposed to act through the cholinergic anti-inflammatory pathway, and is indexed non-invasively by vagally mediated heart rate variability. Disease occurs at a site where local load exceeds available systemic capacity; where load remains within capacity, the site remains healthy. The structure accounts for asymmetric disease (shared capacity, differing local load) and symmetric bilateral disease (a reduction in shared systemic capacity). The autonomic route is presented as one candidate capacity variable among several, alongside redox buffering capacity and vitamin D status, and the predicted association between reduced heart rate variability and peri-implantitis remains untested.

Systemic autonomic capacity sets the threshold. It is a property of the host, shared by every site in the mouth, reflecting the parasympathetic reserve available to restrain the macrophage response wherever it is provoked. Local load determines which sites reach that threshold. It is site-specific and comprises titanium particle burden, biofilm, residual cement, implant position and prosthetic geometry, and occlusal load. In the proposed model, disease develops at a site where local load exceeds the host's available regulatory capacity.

This framework explains the selective case directly: identical hosts but differing local loads, so the most heavily loaded site crosses a shared inflammatory threshold while others do not. It also explains the symmetric case without invoking coincidence: contralateral sites share both similar local loading and the same systemic capacity, so a reduction in regulatory capacity lowers the threshold for both simultaneously, synchronizing outcomes that a purely local account would attribute to chance. It explains the probabilistic behavior of smoking as an additional load and a possible suppressor of host capacity that increases the proportion of sites approaching the threshold without determining any individual outcome. Finally, it explains disease in the absence of conventional risk indicators: a host whose regulatory capacity is reduced, whether associated with chronic stress, dysfunctional breathing, or other causes of reduced vagal tone, in whom ordinary local load is sufficient to trigger disease.

The model's two levels are not always independent. Sympathetic activation and sleep bruxism are mechanistically linked: rhythmic masticatory muscle activity, the physiological signature of sleep bruxism, is consistently preceded by autonomic and respiratory activation, including heart-rate acceleration, increased sympathetic activation, and increased respiratory amplitude, as characterized by Lavigne et al. (59, 60). This activity occurs at a characteristic frequency of approximately 1 Hz (59, 60), comparable to the frequency of physiologic mastication measured directly by Fuentes et al. at 1.46 ± 0.21 Hz (61), but is delivered as sustained, repetitive parafunctional loading rather than the interrupted, food-bolus-modulated cycles of normal chewing. This has direct consequences for the local-load component of the model at implant sites. Bruxism is a recognized risk factor for mechanical implant complications, increasing the odds of prosthetic screw fracture approximately sevenfold and implant fracture more than sixteenfold compared with non-bruxing patients in the meta-analysis by Vidal et al. (62), while Ionfrida et al. reported a pooled odds ratio of 4.68 for implant failure in patients with bruxism (63). Unlike the natural dentition, osseointegrated implants lack periodontal ligament mechanoreceptors and the viscoelastic damping provided by the periodontal ligament, so occlusal forces that a tooth would dissipate through physiologic mobility are instead transmitted more directly to the implant-bone interface, with substantially less physiologic mobility and periodontal ligament damping than at a natural tooth, a comparison quantified by Robinson et al. (64). Conventional titanium is also substantially stiffer than surrounding bone, and this stiffness mismatch alters peri-implant stress and strain transfer under repetitive loading (65). This does not imply that titanium stiffness is independently pathogenic; rather, the material mismatch is one factor determining how parafunctional load is distributed within the local implant-bone system. Reduced autonomic regulatory capacity may therefore act on both levels of the model simultaneously: lowering the threshold at which the shared inflammatory effector is contained while independently increasing the local mechanical load delivered to the site through sympathetically associated parafunctional activity. This is consistent with, and provides a possible mechanism for, the synergy between occlusal load and biofilm already discussed in Part 3, rather than viewing the two as merely coincidence, independent risks.

The model also reframes the surrogate problem of Part 1. Physiologic biologic-width remodeling is the benign component that design factors manage. Once that component is accounted for, what remains is the progressive, inflammatory, host-dependent bone loss that defines the disease. It is precisely this residual component that the design variables fail to predict. The model proposes that systemic regulatory capacity operates within this unexplained residual.

Within this framework, the recurring finding of synergy is expected rather than anomalous. Two loads acting on a gated system interact because each moves the site closer to a threshold determined by the host's regulatory capacity. The framework also accommodates, rather than competes with, other proposed host-capacity variables. Redox buffering capacity, whose failure has been proposed to contribute to peri-implant pathology through oxidative stress and immune dysregulation (66), and vitamin D status, whose insufficiency is associated with higher peri-implant RANKL and lower IL-10 (67), represent additional examples of potentially modifiable systemic host-capacity variables. Autonomic regulatory capacity is proposed as the neural member of this class and the one most directly linked to the macrophage-centered inflammatory effector through the cholinergic anti-inflammatory pathway.

## Part 8. Testable predictions, proposed study, and falsification

A hypothesis earns attention by being testable. This hypothesis generates specific predictions that distinguish it from the prevailing model.

If systemic autonomic regulatory capacity sets the inflammatory threshold, then a measure of that capacity would be expected to predict not only the presence of peri-implantitis but also the number and symmetry of affected sites. Reduced heart rate variability would be predicted to associate with multi-site and more symmetric disease, whereas a patient with preserved heart rate variability who develops peri-implantitis at a single implant would be expected, on investigation, to have an identifiable local cause at that specific site. This is a more specific prediction than the prevailing model currently offers, and it is directly testable.

The proposed first study is a prospective cohort in which heart rate variability is measured before or at implant placement and related to the subsequent incidence of peri-implantitis over a defined follow-up period, with the number and symmetry of affected sites recorded. Potential confounders and covariates affecting HRV or peri-implantitis risk, including age, cardiovascular fitness and physical activity, cardiovascular disease, resting heart rate, medications affecting autonomic function, periodontitis history, smoking, diabetes, supportive maintenance, and relevant local implant factors, should be prespecified and incorporated through standardized measurement, stratification, and multivariable analysis as appropriate. Eligible participants would include adults undergoing dental implant placement who can undergo standardized HRV assessment and longitudinal peri-implant follow-up. Key variables would include baseline vagally mediated HRV indices, resting heart rate, peri-implant clinical and radiographic outcomes, the number and distribution of affected sites, and relevant local implant characteristics. The primary analysis should determine whether the HRV-peri-implantitis association persists after adjustment for relevant demographic, socioeconomic, systemic, behavioral, and local implant-related variables. Cardiovascular disease is considered as one potential covariate because it may influence HRV and is not assumed to have an established causal relationship with peri-implantitis. Because no established pre-clinical population exists for peri-implantitis, an efficient preliminary step would be a cross-sectional study relating heart rate variability to periodontal bone loss in dentate patients. Periodontitis shares the same macrophage–RANKL/OPG inflammatory effector pathway, requires no implants, and provides a practical, low-cost opportunity to establish or exclude an association before undertaking a prospective implant cohort.

The model must also specify what would refute it. Symmetric, multi-site peri-implantitis occurring in a patient with preserved heart rate variability and no identifiable shared local cause would constitute evidence against the hypothesis. A single-site failure with an evident local cause and preserved heart rate variability would be considered neutral rather than supportive. Stating these conditions is essential because a two-level model, applied without discipline, could otherwise accommodate almost any outcome retrospectively.

This detection gap gives the proposed design a structural advantage over the alternatives against which it would be evaluated. Radiographic, ultrasonographic, and biomarker approaches to earlier detection all remain constrained by the same limitation: some local pathological change must already be present before it can be detected. A systemic, pre-clinical measure of regulatory capacity is not subject to this constraint. Heart rate variability measured before implant placement requires no local signal to exist; instead, it characterizes the host's proposed capacity to contain a challenge before disease develops. If validated, it would not compete with earlier detection methods. Rather, it would address a different question by providing a measure of susceptibility instead of attempting to detect disease after local pathology has already begun.

## Part 9. Toward a prevention model

The practical significance of this reframing lies in prevention, where the need for a mechanism-based approach is greatest. A systematic review by Carra et al. of primordial and primary prevention found that no studies had evaluated primordial preventive interventions, and that the evidence supporting the two measures most commonly recommended to patients—smoking cessation and oral hygiene—was inconclusive when assessed as direct preventive interventions for peri-implant diseases (68). For the patient who cannot stop smoking, or who develops disease despite exemplary oral hygiene, the field currently offers no validated strategy aimed specifically at strengthening the host's intrinsic capacity to regulate the inflammatory response.

If autonomic regulatory capacity is a determinant, it is one that can be modified. That distinguishes it from the most extensively studied inherited susceptibility candidate in the field, which is fixed at birth and cannot be changed. Several possibilities follow, each requiring prospective investigation before any clinical application. Heart rate variability could potentially be measured before implant placement as part of future risk stratification, should its predictive value be established. Interventions capable of modifying autonomic regulation, including heart rate variability biofeedback and selected breathing-based approaches, could subsequently be investigated as methods of strengthening this regulatory capacity. These approaches would not replace smoking cessation, glycemic control, supportive maintenance, or other established preventive measures. Rather, they would complement existing prevention by adding a potentially modifiable systemic target for patients in whom conventional preventive strategies are insufficient or cannot be fully implemented. Clinical use of HRV biofeedback and resonance-frequency breathing has been reported in dentistry in the management of TMD and bruxism (69), providing case-level evidence of practical feasibility. However, whether modifying autonomic regulation alters the incidence or progression of peri-implantitis remains entirely untested and requires prospective investigation.

Stimulation of the *α*7 nicotinic acetylcholine receptor with a pharmacological agonist is specifically not proposed. Continuous pharmacological activation is not equivalent to endogenous, dynamically regulated vagal signaling, and sustained activation of *α*7 nicotinic acetylcholine receptors has been reported to suppress bone mass (57). The objective is therefore not continuous receptor activation, but restoration of physiological autonomic regulation through the body's endogenous control mechanisms.

The value of this reframing is straightforward. It does not replace established local preventive strategies; it complements them by adding a proposed systemic dimension focused on the host's regulatory capacity. If validated, it moves prevention upstream, from the implant surface to the regulatory state of the host in whom the implant is placed.

## Limitations

The limitations of this review should be stated explicitly. Its central claim—that autonomic regulatory capacity gates the peri-implant inflammatory response—is not supported by any direct study in implant patients; no published work has linked heart rate variability or vagal tone to peri-implantitis. The mechanistic chain is assembled from adjacent fields, including orthopedic osteolysis, experimental periodontitis, rheumatoid arthritis, and bone neurobiology, and although each link is biologically plausible, its transfer to the peri-implant environment remains unproven. Heart rate variability reflects cardiac parasympathetic modulation and is, at best, an indirect surrogate of the vagal signaling to immune tissues on which the proposed model depends. Direct parasympathetic innervation of the mature human peri-implant bone interface remains incompletely characterized, and the extent to which systemic neuroimmune and non-neuronal cholinergic pathways regulate peri-implant macrophage activity has not yet been established. Likewise, the evidence that the cholinergic anti-inflammatory pathway protects bone derives primarily from inflammatory experimental models, and its effects are context dependent, as discussed above.

The proposed two-level model must also be interpreted within the falsification criteria outlined in Part 8. Without those conditions, it could become insufficiently falsifiable by accommodating disparate clinical outcomes retrospectively. Future studies would also need to address recruitment-related bias, residual confounding, reverse causation, and variability introduced by HRV acquisition conditions. Detailed HRV standardization protocols should be defined prospectively according to the specific study design rather than prescribed within the present hypothesis framework. An additional limitation of the existing evidence is the heterogeneity of peri-implantitis case definitions across studies, which can affect prevalence estimates, reported associations, and comparisons between cohorts. Future prospective testing should therefore use a prespecified and consistently applied diagnostic definition of peri-implantitis. Finally, this is a narrative review rather than a systematic review and is therefore subject to selection bias in the literature considered. Accordingly, the proposed pathway should be regarded as a biologically informed framework for hypothesis generation and prospective testing, rather than as an established mechanism of peri-implantitis.

## Discussion

Peri-implantitis has resisted a satisfactory explanation in part because the surrogate has been measured more often than the disease itself, and because the search for a cause has focused primarily on local loads while the evidence points toward a host-level determinant that has not yet been measured (Figure 3). Early marginal bone loss is substantially physiologic, and the design factors that reduce it have not been shown to reduce peri-implantitis. The factors that do predict disease behave as modifiers acting on a susceptible host, converging on a shared macrophage–NF-*κ*B–RANKL inflammatory effector, while the field's most recent consensus identifies risk indicators rather than an established cause. The most extensively studied inherited host-susceptibility candidate is fixed at birth and cannot be modified, and although the sympathetic stress response has been examined extensively, the parasympathetic counter-regulatory arm has not yet been investigated in peri-implantitis.

**Figure 3:**
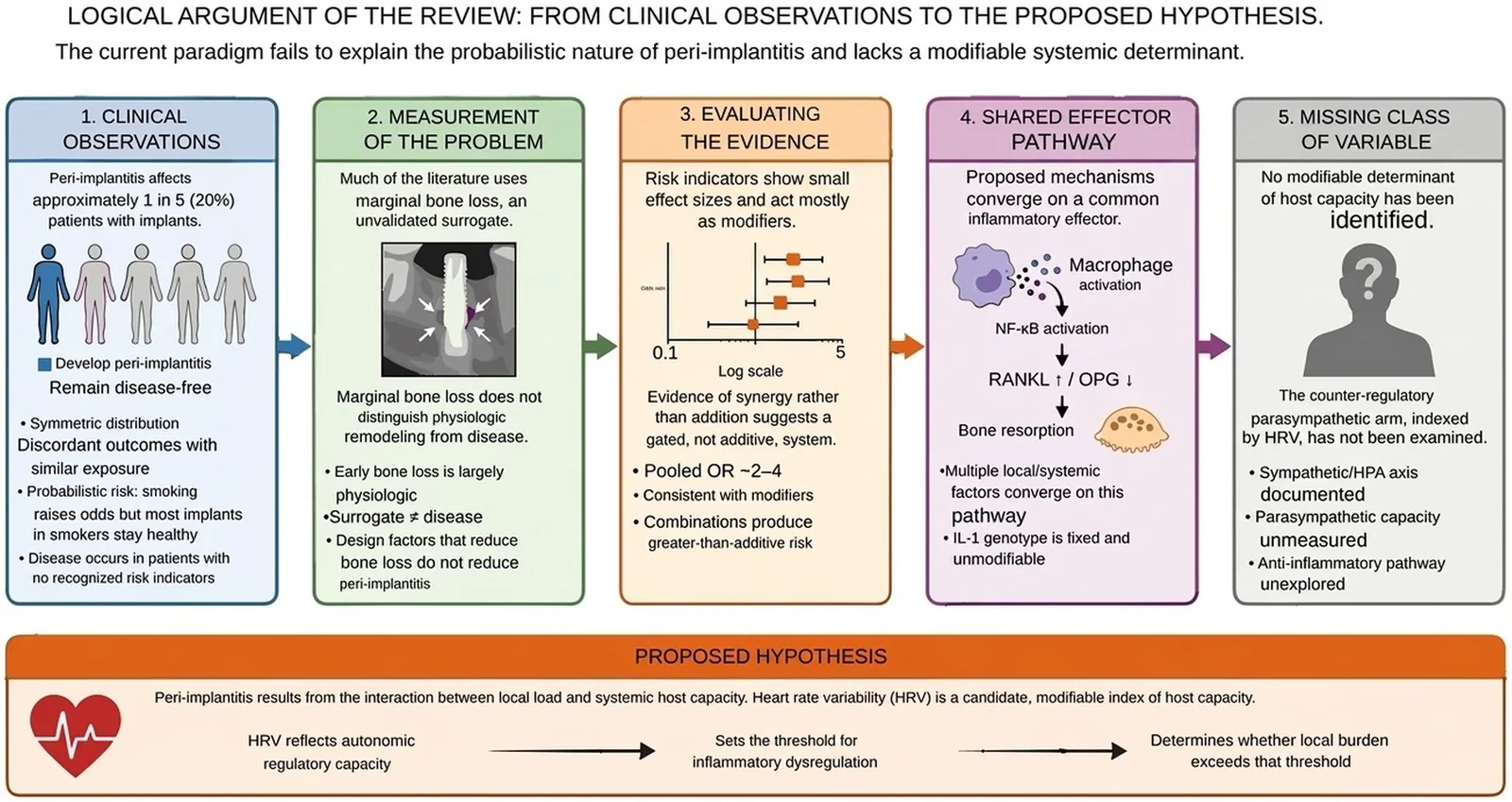
The argument arc, from clinical observations to the proposed hypothesis. A schematic of the review's logic: the clinical scenarios that resist single-cause explanation; the separation of physiological biologic-width remodeling from pathological loss; the sieve showing that design factors predict marginal bone loss but not peri-implantitis while disease predictors are host-level with modifier-magnitude effect sizes; convergence on the shared effector; the resulting gap, a modifiable systemic host-capacity variable, with the sympathetic arm documented and the vagal arm unexamined; and autonomic regulatory capacity proposed to fill it.

This review proposes autonomic regulatory capacity as a candidate modifiable systemic determinant. Its anti-inflammatory influence is proposed to operate, at least in part, through the cholinergic anti-inflammatory pathway, while its cardiac parasympathetic component can be estimated non-invasively using vagally mediated heart rate variability indices. The resulting two-level model proposes that systemic regulatory capacity sets the inflammatory threshold, whereas local load determines which implant sites reach it.

The hypothesis is testable, its predictions are specific, and its conditions for falsification have been stated explicitly. Its immediate priorities are prospective investigation of the relationship between heart rate variability and peri-implantitis and preliminary investigation of its relationship with periodontal bone loss. If supported, this framework would identify autonomic regulatory capacity as a previously untested, modifiable systemic target that complements existing local preventive strategies. More broadly, it would move prevention upstream, from the implant surface to the regulatory state of the host in whom the implant is placed.

## Data Availability

This article presents a theoretical model and literature synthesis with no associated dataset; all cited findings are drawn from previously published, publicly available literature referenced in the article.
